# Periodontitis as a risk factor for colorectal cancer: systematic review and meta-analysis

**DOI:** 10.4317/medoral.27118

**Published:** 2025-04-06

**Authors:** Rosita Elena Espejo-Carrera, Tammy Margarita Honores-Solano, Teresa Verónica Ulloa-Cueva, José Antonio Caballero-Alvarado, Carlos Alberto Minchón-Medina, Angel Steven Asmat-Abanto

**Affiliations:** 1 ORCID ID: 0000-0002-0247-6729. Master of Science in Clinical Research. Professor of the Posgraduate School, Private University Antenor Orrego, Trujillo, Peru. Professor of Stomatology Study Program, Private University Antenor Orrego, Trujillo, Peru; 2 ORCID ID: 0000-0003-0723-3491. Master's degree in Stomatology. Professor of Stomatology Study Program, Private University Antenor Orrego, Trujillo, Peru; 3 ORCID ID: 0000-0003-3334-4777. Doctor in Stomatology. Specialist in Restorative and Aesthetic Dentistry. Professor of Stomatology Study Program, Private University Antenor Orrego, Trujillo, Peru; 4 ORCID ID: 0000-0001-8297-6901. Doctor in Clinical and Translational Research, Specialist in General Surgery. Professor of Human Medicine Study Program, and Posgraduate School, Private University Antenor Orrego, Trujillo, Peru. Physician of Surgery Department, Regional Teaching Hospital of Trujillo, Peru; 5 ORCID ID: 0000-0002-2441-5302. Professor of Faculty of Physical Sciences and Mathematics, Department of Statistics, National University of Trujillo, Peru; 6 ORCID ID: 0000-0001-5726-6692. Doctor in Stomatology. Specialist in Periodontics. Professor of Human Medicine Study Program, Private University Antenor Orrego, Trujillo, Peru. Professor of Stomatology Study Program, Private University Antenor Orrego, Trujillo, Peru. Visiting Professor of the Posgraduate School, University Señor de Sipán, Trujillo, Peru

## Abstract

**Background:**

Primary studies on the association between periodontitis and colorectal cancer (CRC) may have insufficient statistical power to reach a reliable conclusion. In this regard, the present systematic review and meta-analysis were conducted to determine whether periodontitis is a risk factor for CRC.

**Material and Methods:**

A systematic search was carried out in five databases, which included cohort and case-control studies published up to July 3, 2024, in which periodontitis was evaluated as a risk factor for CRC using relative risk (RR), hazard ratio (HR) or odds ratio (OR). The Newcastle-Ottawa Scale (NOS) was used to assess the risk of bias, and the GRADE system was used to determine the certainty of the evidence.

**Results:**

Of 1476 articles retrieved, 8 cohort studies were included for qualitative analysis and meta-analysis. The overall synthesis showed that periodontitis is not a risk factor for CRC (RR=1.34; 95% CI: 0.96-1.89; *p*<0.09; I2=95%). In addition, subgroup analyses were performed according to gender, periodontitis diagnostic methods, and risk of bias, which led to the finding of an increased risk of CRC of 32% only for men with periodontitis (RR=1.32; 95% CI: 1.16-1.50; *p*<0.00001; I2=0%).

**Conclusions:**

Periodontitis is not a risk factor for CRC, with very low certainty of evidence. However, the analysis of subgroups by gender showed that it is a risk factor for CRC in men, with moderate certainty of evidence.

** Key words:**Periodontitis, periodontal diseases, colorectal neoplasms, colorectal cancer, colorectal carcinoma, colorectal tumor.

## Introduction

Periodontitis, one of the most prevalent inflammatory diseases, causes progressive and irreversible destruction of periodontal tissues, which eventually leads to mobility and tooth loss. The most serious condition of this disease affects between 10.8 and 11.2% of the world's population (1,2).

Colorectal cancer (CRC) is one of the leading causes of morbidity and mortality worldwide, and it is ranked second as regards the number of deaths associated with malignant neoplasms in both men and women. Furthermore, it is the third most commonly diagnosed form of cancer, accounting for approximately 10% of all new cases of cancer (3-6).

Numerous pathophysiological mechanisms, such as abnormal cell proliferation, cell differentiation, resistance to apoptosis, invasion of adjacent structures, and metastasis, are involved in colorectal carcinogenesis (4). These processes are initiated by the complex interaction of genetic and environmental factors, including sedentary lifestyle, obesity, alcohol consumption, smoking, and intestinal microbiota (3,4). Primary and secondary prevention and education about risk factors are essential elements for reducing the burden of this disease and improving prognosis and survival rates (5,7). Although there are systemic treatments, the rapid progress of the disease and its resistance to existing treatments have generated the need to investigate its risk factors, progression, and resistance to therapy (6).

The interaction of cancer cells with the microbiota is a unique feature in CRC compared to other types of cancer. Under normal conditions, the microbiota plays a protective role by participating in immunological homeostasis and the growth of intestinal lymphatic tissues; however, a change in balance leads to the proliferation of opportunistic pathogenic bacteria (6). In the cases of CRC, two specific species of periodontal bacteria are outstanding, namely *F. nucleatum* and *P. *gingivalis**; the former has been more noTable in regional lymph node metastases (8). These pathogens produce harmful metabolites, including toxins and antigens, that cause maladaptive immune responses and inflammation, damage to DNA, genomic instability, and tumor progression (6).

Translocation of periodontal pathobionts with oncogenic properties and subgingival proinflammatory mediators could potentiate carcinogenic agents, thereby changing the conditions favoring the development and progression of CRC (6,9).

Despite the growing evidence that periodontitis could be a risk factor for CRC, the primary studies may have insufficient statistical power to reach a reliable conclusion. In this sense, the present systematic review and meta-analysis were conducted to evaluate the possible association between periodontitis and the risk of CRC.

## Material and Methods

- Protocol and registration

The present systematic review was carried out according to the Preferred Reporting Items for Systematic Reviews and Metanalyses checklist (PRISMA, 2020) (10), and the protocol was registered in PROSPERO under the reference number CRD42024557822.

- Focused question

The research question: Is periodontitis a risk factor for colorectal cancer in adult patients? The question was raised according to the PECOS search strategy (population/patients, exposure, comparison, results, and design of study), where *P* = adults, E = exposure to periodontitis, C = absence of periodontitis, O = risk of colorectal cancer, and S = cohort and case-control studies.

- Eligibility criteria and process of selection

The observational cohort design and case-control studies included published until July 3, 2024, which studied periodontitis as a risk factor for CRC in adult patients (Over 18 years of age) and calculated the odds ratio (OR), relative risk (RR) or hazard ratio (HR). The excluded studies did not have an independent statistical analysis of the risk of CRC, those with incomplete data, fewer than 1000 participants, and a follow-up period of less than 5 years.

- Operational definitions

Periodontitis was diagnosed using clinical, radiographic assessment, and epidemiological periodontal indicators. Hospital records and self-reports were also considered.

- Search strategy

The search was carried out in the electronic databases of PubMed/Medline, Web of Science, Scopus, Embase, and BVS, as well as manual searches in the reference lists of all included studies and revisions previously published. The complete search strategy, adapted according to the syntax rules of each database, is presented in Supplement 1.

- Data extraction and synthesis

The bibliographic search results were uploaded to the Rayyan application for systematic revisions (https://www.rayyan.ai/), and the duplicate records were eliminated. Two researchers (T.H.S. and T.U.C.) independently selected the articles to be analyzed, first by title and abstract, then four researchers working in pairs (T.H.S. with T.U.C. and R.E.C with A.A.A) analyzed the complete texts. Any disagreement was discussed among the above-mentioned researchers. Therefore, the data was independently extracted and entered into an Excel spreadsheet (Microsoft® Excel® for Office 365). Afterward, the articles selected and data extracted were revised and approved by a fifth expert investigativ e (J.C.A.).

- Risk of bias and certainty of evidence

The studies included were analyzed using the Newcastle-Ottawa Scale (NOS) tool to assess the risk of bias. The quality of evidence of the studies included in the meta-analysis was evaluated by the GRADE tool, using the GRADEpro GDT software (11).

- Statistical analysis

Data collected from the studies selected for the meta-analysis were processed using RevMan software (Review Manager v.5.3, The Cochrane Collaboration) to evaluate periodontitis as a risk factor for CRC. The random effects model was applied to develop the forest plot, and the heterogeneity between the studies was assessed using the I2 index. A sensitivity analysis was also performed to verify each study's influence on the pooled results. Subgroup analysis was performed to determine potential sources of variability. Moreover, an assessment of potential publication bias was performed using Egger's test and Stata 16.0 software (StataCorp LLC, College Station, TX).

- Summary of results

The primary outcome was periodontitis as a risk factor for CRC. The information required from each study was collected in a preliminary summarized Table, and results with sufficient data to calculate an effect estimate were used for the meta-analysis.

## Results

- Selection of studies

**Figure 1:**
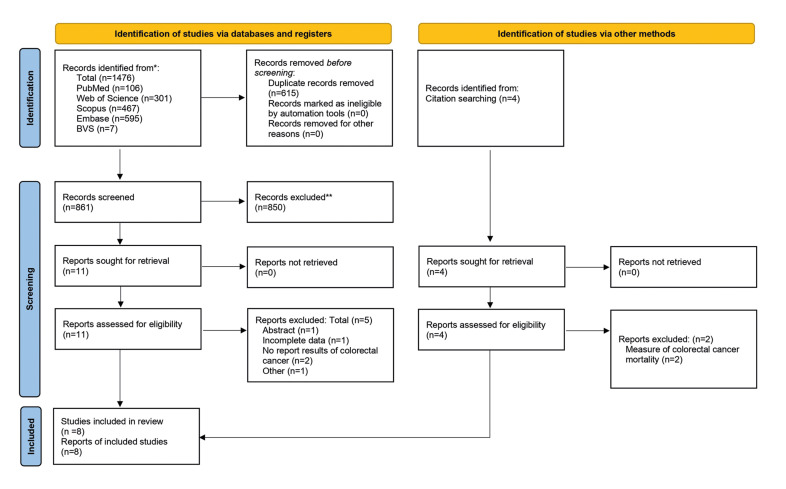
Flow chart for inclusion of the studies evaluating periodontitis as a risk factor for CRC.

As presented in the PRISMA 2020 flowchart (12) (Fig. 1), 1476 records were retrieved. After removing duplicates and screening by title and abstract, 11 articles (10 cohort and one case-control study) remained for full-text evaluation, of which 5 (4 cohort and one case-control) were excluded. After manually searching the references of previous systematic reviews, four additional studies were retrieved, of which two were excluded. The reasons for excluding studies are described in detail in Supplement 2. Finally, eight studies were chosen for qualitative analysis and meta-analysis.

- Characteristics of studies

In the 8 cohort studies included (Table 1), 988,897 subjects were evaluated; the study with the smallest number of participants had 1,337 (1,022 exposed and 315 unexposed) (13), and the one with the largest number had 713,201 (53,075 exposed and 660,126 unexposed) (14). The ages of the patients studied ranged from 15 (14) to 85 years (13); the shortest follow-up period was 10 years (14), and the longest was 41 years (15).

The included studies were conducted in the United States (13,16-20), Sweden (15), and Korea (14). All studies declared that they had been funded; while five (14-17,19) mentioned that there was no conflict of interest, two (13,20) did not mention it, and one (18) indicated that the financing agent participated in the design and data collection.

- Risk of bias

As shown in Table 2, most of the studies included in the present analysis had a low risk of bias (13,14,16,17,19,20). The NOS categories with the lowest compliance were the representativeness of the cohort exposed and the determination of exposure.

- Meta-analysis of synthesis

**Figure 2:**
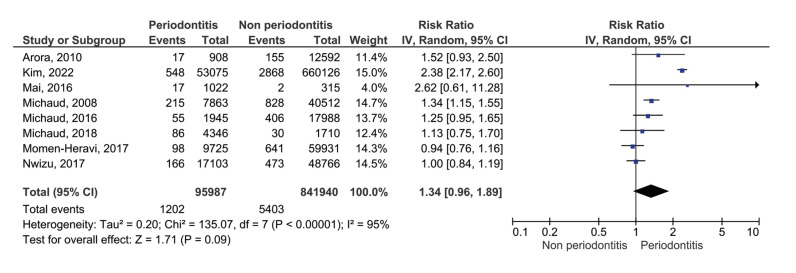
Forest plot of the studies evaluating periodontitis as a risk factor for CRC.

The 8 cohort studies were analyzed using the random effects model and considering the relative risk (RR) as the measure of effect. The global synthesis showed a RR of 1.34 (95% CI: 0.96-1.89) and *p*=0.09, with considerable general heterogeneity (I2=95%; *p*<0.00001), as shown in the forest plot of Fig. 2. In this sense, because the CI crosses the line of no effect, periodontitis would not be a risk factor for CRC.

- Analysis of subgroups

Analysis of the subgroups, which included gender, methods of diagnosis of periodontitis, and risk of bias, was performed, as shown in Table 3. The heterogeneity drastically decreased in the subgroup gender in the studies that included only men (I2=0%, *p*=0.68), only women (I2=0%, *p*=0.38), and an unclear risk of bias (I2=0%, *p*=0.36). A decrease in heterogeneity, although moderate, was also found when the method of periodontitis diagnosis was self-reported (I2=59%, *p*=0.12). In this sense, the analysis showed an increase of 32% in the CRC risk for men with periodontitis (RR=1.32; 95% CI: 1.16-1.50; *p*<0.00001). The forest plot of the analysis of subgroups may be visualized in Supplemen 3t.

- Publication bias and sensitivity analysis

The Egger test was used, and no publication bias (t=0.40, *p*=0.7026) was detected, which was confirmed by the Harbor test (t=0.21, *p*=0.8407) and the Begg test based on the Kendall score (z=1.11, *p*=0.2655).

The sensitivity analysis is shown in Table 4, in which the study of Momen-Heravi *et al*. (16) was omitted. An association was found between periodontitis and CRC (RR=1.43; 95%CI:1.00-2.03; *p*=0.05). Moreover, omitting the study of Kim *et al*. (14) decreased the heterogeneity to 53%. However, the decision was to maintain these studies due to the statistical power of the sample.

- Quality of evidence

**Figure 3:**
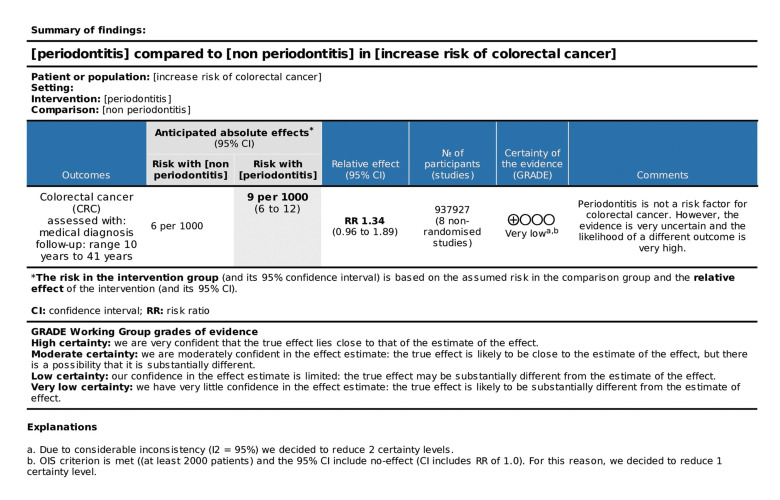
SoF table of the eight studies evaluating periodontitis as a risk factor for CRC.

**Figure 4:**
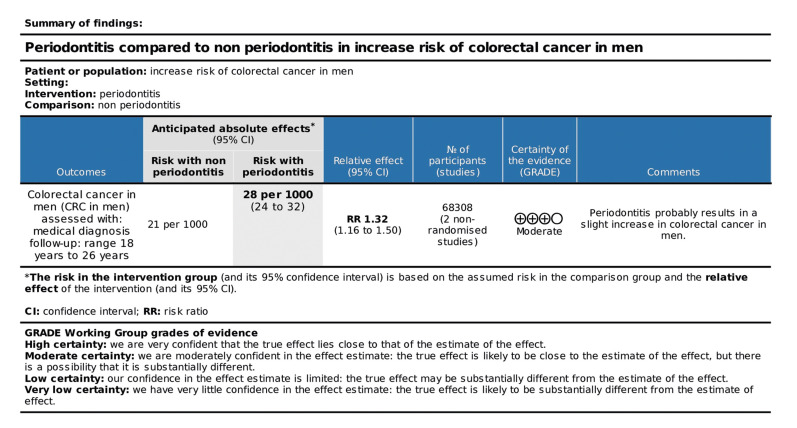
SoF table of the studies evaluating periodontitis as a risk factor for CRC in men.

As presented in the SoF Table in Fig. 3, periodontitis was not a risk factor for CRC, with very low certainty of evidence, indicating that the probability of a different result for future studies would be very high. Considering the finding that periodontitis was a risk factor for CRC in the male gender, a second GRADE analysis was performed (Fig. 4) with the studies included in this subgroup, obtaining a moderate certainty of evidence, which suggested a good indication of the probable effect.

## Discussion

In recent years, studies have suggested a link between periodontitis and the risk of gastrointestinal cancer. The exact biological mechanisms of this association have not yet been fully understood. Some potential pathways have, however, been identified, such as the production of carcinogenic metabolites by specific oral microorganisms and the induction of chronic low-grade systemic inflammation, leading to the release of free radicals and cytokines (21).

Although the overall synthesis showed an RR of 1.32, in the present meta-analysis, the CI crossed the line of no effect, indicating that periodontitis was not a risk factor for CRC. This was in disagreement with the findings of Wang *et al*. (21) and Li *et al*. (22), who reported that periodontal disease (PD) increased the risk of CRC by 21% and 44%, respectively. These authors used HR as a measure of effect, and although this can be interpreted as an RR, it can convey different information. The former quantifies the instantaneous relative risk, while RR quantifies the cumulative risk; therefore, these two estimates may differ considerably for the same data (23). It should be noted that the meta-analysis by Wang *et al*. included one study (24) that did not evaluate CRC directly; instead, it evaluated benign and malignant colorectal tumors and found an association only for the former. Likewise, the review by Li *et al*. included one study (25) that evaluated mortality. In contrast, both reviews evaluated PD (gingivitis and periodontitis) and not specifically periodontitis, accentuating the discrepancy.

When performing subgroup analysis, a 32% increase in the risk of CRC was found for men with periodontitis, coinciding with the findings reported by Li *et al*. (22). This is due to the differences in exposure to risk factors such as alcohol and tobacco, dietary patterns and sex hormones. Considering that men diagnosed with CRC had a worse prognosis and a 40% higher mortality rate than women (26), strategies for early detection of periodontitis and colonic lesions with oncogenic potential should be considered for this group.

A limitation to be considered for the present review would be the high heterogeneity, possibly due to the large difference in sample sizes, population characteristics, criteria for classifying exposed/non-exposed participants, and indicators for periodontitis diagnosis, among other factors. Concerning sample sizes, the study by Mai *et al*. (13) had a sample of only 1337 participants, while Kim *et al*. (14), Momen-Heravi *et al*. (16), and Nwizu *et al*. (17) had samples larger than 50,000 participants, which would contribute to the power of the meta-analysis for detecting real effects (27-29). Similarly, the study by Kim *et al*. (14) showed a significant difference in the ages of their exposed vs. unexposed groups, with mean ages of 49 [39-60] and 31 [15-46], respectively. This difference in ages could be critical, considering that CRC occurs more frequently in patients over 50 years of age (26,30), and the approximate period in which malignancy findings may occur in the colorectal region is 10 and 15 years, mainly in cases of polyps or in patients with Lynch syndrome (26,31). Moreover, concerning the diagnosis of periodontitis, the majority of studies included in this analysis were based on self-reports validated in previous studies by radiographic bone loss or clinical examination. Only a few used a periodontal index or clinical and/or radiographic diagnosis, the latter being, according to Heitz (32), the standard method for periodontitis diagnosis. While it is true that the use of self-reported data could be susceptible to recall bias or patients could modify their responses to align with concepts they considered socially accepTable, an interesting finding of the present systematic review and meta-analysis was that the self-report subgroup data did not differ from those of the clinical-radiographic diagnosis of periodontitis, which would suggest that self-report could be a reliable method for use in this type of studies.

According to the GRADE assessment of the eight studies included in the meta-analysis, there was great uncertainty in the results, and further studies on the subject would likely provide substantially different results. However, when assessing studies that included men, a moderate certainty of evidence was obtained, indicating that the results probably reflect the situation that occurred in reality. From this aspect, we do recommend that future studies must focus on the presence or predominance of bacterial complexes with evidence of oncogenic properties, such as *Fusobacterium nucleatum* and *Porphyromonas *gingivalis** (33-35), and show specific results according to gender. Likewise, the periodontal epidemiological index should be homogenized to classify the exposed group and incorporate the evaluation of biomarkers of chronic systemic inflammation.

## Conclusions

Periodontitis is not a risk factor for CRC, with very low certainty of evidence and high heterogeneity of the studies included. However, periodontitis is a risk factor in males, with moderate certainty of evidence and without heterogeneity.

## Figures and Tables

**Table 1:** General characteristics of cohort studies included.

Nº	Author (country, year of publication)	Database	Design	Follow-up period (years)	Age (Years)	No. of Participants		The primary location of colorectal cancer	Periodontal characteristic	Covariables	Conclusions
						Exposed	Non-exposed				
1	Kim (Korea, 2022)	Korean National Health Insurance Cohort Database	Retrospective	10	Periodontitis: 49 (39-60) Controls: 31 (15-46)	Total: 53075 Men: 27310Women: 25765	Total: 660126 Men: 325796 Women: 334330	Colon	Community periodontal index: Code 3: presence of moderate pocket (4-5 mm) Code 4: Deep pocket (probing equal to or greater than 6 mm)	Age, sex, level of income, residential areas, BMI, smoking history, and comorbidities (MI, CHF, PVD or rest pain, any history of CVA, dementia, COPD, CTD, PUD, DM, moderate to severe CKD, hemiplegia, leukemia, malignant lymphoma, ascites or esophageal varices, disseminated cancer, and AIDS)	Periodontitis was associated with an increased risk of cancer. Diagnosis of periodontitis was associated with an increased risk of colon cancer.
2	Momen-Heravi (USA, 2017)	Nurses' Health Study	Prospective	18	30 - 55	Total: 9725 Women: 9725 Men: 0	Total: 59931 Women: 59931 Men: 0	Colorectal, colon and rectum	Self-reported periodontitis	Age, race, alcohol consumption, intakes of folate, calcium, vitamin D, red meat and processed meat, multivitamin use, BMI, physical activity, cigarette smoking, history of sigmoidoscopy/ colonoscopy screening, family history of CRC, aspirin use, Type 2 DM and PMH use.	Women with fewer teeth and possibly moderate or severe bone loss may have a slightly higher risk of developing colorectal cancer, suggesting a possible role of oral health in colorectal carcinogenesis.
3	Michaud (USA, 2018)	Atherosclerosis Risk in Communities (ARIC cohort study)	Prospective	14.7	44 - 66	Total: 4346 The data provided were expressed in percentages without specifying the total number of men and women separately.	Total: 1710 The data provided were expressed in percentages without specifying the total number of men and women separately.	Colorectal	Probing depth and clinical attachment loss (CAL);	Age, race, educational status, field center, BMI, DM, HRT in women smoking and drinking status.	The risk of cancer, especially lung and colorectal cancer, was higher in individuals with periodontitis.
4	Michaud (USA, 2016)	Health Professionals Follow-up Study (HPFS)	Prospective	26	No PD and 17-32 teeth: 52.2 (±9.56); No PD and <17 teeth: 61.3 (±9.20); PD and 17-32 teeth 56.5 (±9.59); PD y <17 teeth: 64.8 (±8.06)	Total: 1945 Men: 1945 Women: 0	Total: 17988 Men: 17988 Women: 0	Colorectal	Self-report of periodontitis	Age, height, BMI, physical activity, ethnic origin, DM, high cholesterol, high blood pressure, NSAID, multivitamin, daily dietary intake (calories, vitamin D, alcohol)	Periodontitis was not associated with colorectal cancer.
5	Arora (Sweden, 2010)	The Swedish Twin Registry	Prospective	41	No PD: 50.6 (±9.4); PD: 58.7 (±9.7)	Total: 908 The data provided were expressed in percentages without specifying the total number of men and women separately.	Total: 12592 The data provided were expressed in percentages without specifying the total number of men and women separately.	Colorectal	Self-reported periodontitis: If at least half of the patient's teeth showed mobility, this was indicative of advanced disease.	Sex, age, education, employment, number of siblings, smoking status, smoking status of partner, alcohol status, BMI, DM	Periodontitis was associated with an increased risk of colorectal cancer.
6	Michaud (USA, 2008)	Health Professionals Follow-up Study (HPFS)	Prospective	18	No PD: 53.7 (±9.7); PD: 58.3 (±9.2)	Total: 7863 Men: 7863 Women: 0	Total: 40512 Men: 40512 Women: 0	Colorectal	Self-report of periodontitis	Age, height, BMI, physical activity, race, smoking history, disease history (DM, Cholecystectomy), medication use (aspirin, NSAID, multivitamin) daily dietary intake (calories, vitamin C, vitamin D, alcohol).	No association was found between periodontitis and colorectal cancer.
7	Mai (USA, 2016)	Women´s Health Initiative (WHI) Observational	Prospective	12.2	53 - 85	Total: 1022 Men: 0 Women: 1022	Total: 315 Men: 0 Women: 315	Colorectal	Radiographic assessment: loss of alveolar ridge height.	Age at the visit, race, the highest level of education, smoking history, secondhand exposure to smoke, alcohol consumption, physical activity, total energy intake, fruit and vegetable intake, red meat intake, caffeine intake, lactose intake, hormone use plus progesterone, current regular use of NSAID, current dietary supplement use (any vitamin, calcium, and vitamin D), history of diagnosis of DM, dental hygiene (frequency of toothbrushing, tooth flossing, and dental visits), family history of cancer, age at menopause, age at menarche, parity, and use of oral contraceptives, BMI, smoking history included smoking status, packs smoked per day, age when the patient started smoking.	There were no statistically significant associations between the severity of periodontitis and the risk of total or site-specific cancer.
8	Nwizu (USA, 2017)	Study at the Buffalo Clinical Center	Prospective	15	50 - 79 Mean: 68.3	Total: 17103 Men: 0 Women: 17103	Total: 48766 Men 0 Women: 48766	Colorectal, colon and rectum	Self-report of history of PD	Age, race/ethnicity, educational level, region of residence, family history of cancer, history of DM, recreational physical activity, smoking status, pack-years of smoking, exposure, pt secondhand smoke, alcohol consumption, total dietary energy intake, fruit and vegetable intake, total (dietary & supplement) intake of calcium and vitamin D and MHT use, frequency of dental visits, BMI.	No association was found between PD and colorectal cancer.

Abbreviations: PD = periodontal disease, CRC = colorectal cancer MI = myocardial infarction, CHF = congestive heart failure, PVD = peripheral vascular disease, CVA = cerebrovascular accident, CTD = connective tissue disease, PUD = peptic ulcer disease, CKD = chronic kidney disease, BMI = body mass index, HRT = hormone replacement therapy, DM = diabetes mellitus, COPD = chronic obstructive pulmonary disease, AIDS = acquired immune deficiency syndrome, PMH = post-menopausal hormone, NSAID = non-steroidal anti-inflammatory drug, MHT = menopausal hormone therapy.

**Table 2:** Summary of the risk of bias assessment for cohort studies - New Castle Ottawa.

Study	Selection				Comparability	Outcome			NOS
	Representativeness of the exposed cohort	Selection of the non-exposed cohort	Ascertainment of exposure	Demonstration that outcome of interest was not present at the start of the study	Comparability of cohorts based on the design or analysis	Assessment of outcome	Was follow-up long enough for outcomes to occur?	Adequacy of follow-up of cohorts	
Kim, 2022	*	*	*	*	**	*	*	*	9
Momen-Heravi, 2017	-	*	*	*	**	*	*	*	8
Michaud 2018	-	*	*	-	**	*	*	-	6
Michaud 2016	-	*	-	*	**	*	*	*	7
Arora, 2010	-	*	-	*	**	*	*	-	6
Michaud 2008	-	*	-	*	**	*	*	*	7
Mai, 2016	-	*	*	*	**	*	*	*	8
Nwizu, 2017	-	*	-	*	**	*	*	*	7

**Table 3:** Results of subgroup analysis.

Subgroups		No. of studies	Heterogeneity		Effect model	Meta-analysis			Meta-regression
			I^2^(%)	*p*		RR	95% CI	*p*	
Gender	Male	2	0	0.68	Random	1.32	1.16-1.50	< 0.00001	0.004
	Female	3	0	0.38	Random	0.98	0.86-1.13	0.82	
	Male and female	3	86	0.0007	Random	1.65	0.98-2.76	0.06	
Method of periodontitis diagnostic	Clinical-radiographic	6	95	< 0.00001	Random	1.4	0.94-2.07	0.09	0.500
	Self-report	2	59	0.12	Random	1.32	0.97-1.79	0.47	
Risk of bias	Low risk of bias	6	96	< 0.00001	Random	1.36	0.91-2.03	0.13	0.800
	Unclear risk of bias	2	0	0.36	Random	1.27	0.93-1.75	0.13	

**Table 4:** Sensitivity analysis.

Omited study	No. of studies	Heterogeneity		Meta-analysis		
		I^2^(%)	*p*	RR	95% CI	*p*
Arora, 2010	7	96	< 0.00001	1.32	0.92-1.91	0.13
Kim, 2022	7	53	0.05	1.16	0.99-1.35	0.06
Mai, 2016	7	96	< 0.00001	1.31	0.92-1.85	0.13
Michaud, 2008	7	95	< 0.00001	1.35	0.89-2.05	0.16
Michaud, 2016	7	95	< 0.00001	1.36	0.93-1.99	0.11
Michaud, 2018	7	95	< 0.00001	1.38	0.96-1.99	0.09
Momen-Heravi, 2017	7	94	< 0.00001	1.43	1.00-2.03	0.05
Nwizu, 2017	7	94	< 0.00001	1.41	0.99-2.02	0.06
